# Blood-brain barrier-associated pericytes internalize and clear aggregated amyloid-β42 by LRP1-dependent apolipoprotein E isoform-specific mechanism

**DOI:** 10.1186/s13024-018-0286-0

**Published:** 2018-10-19

**Authors:** Qingyi Ma, Zhen Zhao, Abhay P Sagare, Yingxi Wu, Min Wang, Nelly Chuqui Owens, Philip B Verghese, Joachim Herz, David M Holtzman, Berislav V Zlokovic

**Affiliations:** 1 Center for Neurodegeneration and Regeneration, Zilkha Neurogenetic Institute and Department of Physiology and Neuroscience, Keck School of Medicine, University of Southern California, Los Angeles, California 90033 USA; 2 Lawrence D. Longo, MD Center for Neonatal Biology, Division of Pharmacology, Department of Basic Sciences, Loma Linda University School of Medicine, Loma Linda, CA 92350 USA; 3 C2N Diagnostics, LLC, Saint Louis, MO 63110 USA; 4 Department of Molecular Genetics, University of Texas Southwestern Medical Center, Dallas, TX USA; 5 Department of Neuroscience, University of Texas Southwestern Medical Center, Dallas, TX USA; 6 Department of Neurology and Neurotherapeutics and Center for Translational Neurodegeneration Research, University of Texas Southwestern Medical Center, Dallas, TX USA; 7 Department of Neurology, Hope Center for Neurological Disorders, Knight Alzheimer’s Disease Research Center, Washington University School of Medicine, Saint Louis, MO 63110 USA

**Keywords:** Pericyte, Blood-brain barrier (BBB), Amyloid-β clearance, Low-density lipoprotein receptor-related protein 1 (LRP1), Apolipoprotein E

## Abstract

**Background:**

Clearance at the blood-brain barrier (BBB) plays an important role in removal of Alzheimer’s amyloid-β (Aβ) toxin from brain both in humans and animal models. Apolipoprotein E (apoE), the major genetic risk factor for AD, disrupts Aβ clearance at the BBB. The cellular and molecular mechanisms, however, still remain unclear, particularly whether the BBB-associated brain capillary pericytes can contribute to removal of aggregated Aβ from brain capillaries, and whether removal of Aβ aggregates by pericytes requires apoE, and if so, is Aβ clearance on pericytes apoE isoform-specific.

**Methods:**

We performed immunostaining for Aβ and pericyte biomarkers on brain capillaries (< 6 μm in diameter) on tissue sections derived from AD patients and age-matched controls, and *APP*^*Swe/0*^ mice and littermate controls. Human Cy3-Aβ42 uptake by pericytes was studied on freshly isolated brain slices from control mice, pericyte LRP1-deficient mice (*Lrp*^*lox/lox*^*;Cspg4-Cre*) and littermate controls. Clearance of aggregated Aβ42 by mouse pericytes was studied on multi-spot glass slides under different experimental conditions including pharmacologic and/or genetic inhibition of the low density lipoprotein receptor related protein 1 (LRP1), an apoE receptor, and/or silencing mouse endogenous *Apoe* in the presence and absence of human astrocyte-derived lipidated apoE3 or apoE4. Student’s t-test and one-way ANOVA followed by Bonferroni's post-hoc test were used for statistical analysis.

**Results:**

First, we found that 35% and 60% of brain capillary pericytes accumulate Aβ in AD patients and 8.5-month-old *APP*^*Sw/0*^ mice, respectively, compared to negligible uptake in controls. Cy3-Aβ42 species were abundantly taken up by pericytes on cultured mouse brain slices via LRP1, as shown by both pharmacologic and genetic inhibition of LRP1 in pericytes. Mouse pericytes vigorously cleared aggregated Cy3-Aβ42 from multi-spot glass slides via LRP1, which was inhibited by pharmacologic and/or genetic knockdown of mouse endogenous apoE. Human astrocyte-derived lipidated apoE3, but not apoE4, normalized Aβ42 clearance by mouse pericytes with silenced mouse apoE.

**Conclusions:**

Our data suggest that BBB-associated pericytes clear Aβ aggregates via an LRP1/apoE isoform-specific mechanism. These data support the role of LRP1/apoE interactions on pericytes as a potential therapeutic target for controlling Aβ clearance in AD.

**Electronic supplementary material:**

The online version of this article (10.1186/s13024-018-0286-0) contains supplementary material, which is available to authorized users.

## Background

Alzheimer’s disease (AD) is a progressive neurodegenerative disorder associated with cognitive impairment, early neurovascular changes, accumulation of amyloid-β (Aβ) and tau pathology, and neuron loss [1, 2]. According to the amyloid hypothesis, the build-up of Aβ in the brain parenchyma [3–5] and blood vessels [6–9] is the key event leading to other AD-related pathologies and disease symptoms. In the brain, Aβ is produced by neurons and other cell types, and is constantly removed by several clearance mechanisms. This includes receptor-mediated transport across the blood-brain barrier (BBB) into the peripheral circulation [10], enzyme-mediated Aβ proteolytic degradation [11], removal by glial cells [12], and diffusive transport across the interstitial fluid (ISF) along the perivascular spaces [13–15] leading to drainage by the meningeal lymphatic system [16, 17]. The imbalances between Aβ production and clearance result in Aβ deposition in the brain [18]. Kinetic studies in patients diagnosed with sporadic AD indicate that faulty Aβ clearance, rather than Aβ overproduction, is critical for accumulation of Aβ in the brain [19]. Moreover, recent transport studies in humans have shown that up to 50% of the Aβ in the brain is transported across the BBB to blood [20], confirming prior findings in animal models [21, 22].

The endothelial cells of the BBB, and the BBB-associated mural cells - pericytes and vascular smooth muscle cells (VSMCs), and glial cells clear Aβ, which can lead to Aβ transport across the BBB to blood, and/or Aβ degradation by mural cells, astrocytes and/or microglia [10, 18]. The low-density lipoprotein receptor-related protein 1 (LRP1), an apolipoprotein E (apoE) receptor [23, 24], mediates internalization of soluble Aβ at the abluminal side of the BBB [22, 25, 26]. This is followed by Aβ transcytosis across the BBB that is regulated by PICALM (Phosphatidylinositol Binding Clathrin Assembly Protein) and Rab5 and Rab11 small GTPases, ultimately leading to Aβ exocytosis across the luminal side of the BBB and clearance into the blood [27]. Consequently, endothelial-specific deletion of *Lrp1* gene [28] or deletion of *Picalm* gene from the endothelium [27] lead to accelerated Aβ pathology in Aβ-precursor protein (*APP*) overexpressing mice. VSMCs within the small cerebral arteries also clear Aβ via LRP1 [29, 30]. Similarly, astrocytes clear deposited Aβ via LRP1, which requires mouse endogenous apoE [31].

Brain capillary pericytes are centrally positioned between brain endothelial cells, astrocytes and neurons [32]. Besides roles in regulating BBB permeability [33, 34] and cerebral blood flow [35], pericytes show strong phagocytic activity associated with clearance of toxic foreign molecules [32], and endogenous proteins [36] including Alzheimer’s Aβ, which can influence development of Aβ pathology as shown in *APP* mouse models [37, 38]. Prior studies have demonstrated that apoE disrupts Aβ clearance across the mouse BBB [39], specifically, apoE4, which carries major genetic risk for AD [40–42], had greater disruptive effect than apoE3, which carries lower risk. However, the contribution of BBB-associated pericytes compared to endothelial trans-vascular transport to Aβ clearance at the BBB still remains elusive, particularly, whether pericytes can contribute to removal of aggregated Aβ from brain capillaries, which develop cerebral amyloid angiopathy (CAA) in AD [8], and whether removal of Aβ aggregates by pericytes requires apoE, and if so, is Aβ clearance on pericytes apoE isoform-specific? Here we show that Aβ accumulates in abundance in brain pericytes in AD and *APP*^*Sw/*0^ mice, suggesting their active role in Aβ removal at the BBB. Moreover, we show that pericytes internalize and clear Aβ aggregates by an LRP1/apoE isoform-specific mechanism implying that targeting LRP1/apoE pathway in pericytes has potential to control Aβ clearance in AD.

## Methods

### Animals

Mice were housed in plastic cages on a 12 h light cycle with ad libitum access to water and a standard laboratory diet. All procedures were approved by the Institutional Animal Care and Use Committee at the University of Southern California with National Institutes of Health guidelines. We studied *APP*^*Sw/0*^ mice expressing human APP transgene with the K670 M/N671 L (Swedish) double-mutation under control of the hamster prion promoter [43]. *Lrp*^*lox/lox*^ mice [44, 45] were crossed with *Cspg4-Cre* mice [46] to generate *Lrp*^*lox/lox*^*;Cspg4-Cre* mice with deletions of *Lrp1* gene from pericytes and oligodendrocyte progenitor cells. To minimize confounding effects of background heterogeneity all experiments were performed using age-matched littermates. Both, male and female mice were used in the study. All animals were randomized for their genotype information. All experiments were blinded; the operators responsible for experimental procedure and data analysis were blinded and unaware of group allocation throughout the experiments.

### Human tissue immunofluorescence analysis

Written consent was obtained and approved by the University of Rochester Medical Center for all studied human subjects prior to death. The postmortem interval ranged between 4 and 16 h. Postmortem brain tissue samples including frontal cortex (Brodmann area 9/10) were obtained from subjects with a definite diagnosis of AD confirmed by neuropathological analysis including Braak stages ≥ III; CERAD (Consortium to Establish a Registry for Alzheimer’s Disease) frequent, and neurologically intact controls with no AD pathology (Braak stages ≤ III; CERAD – negative). Six controls and six AD samples were used for the current study. Their demographic and clinical features were as we reported previously [47], and shown in Additional file 1: Table S1. Vascular risk factors such as atherosclerosis, hypertension and/or myocardial infarction were present in 4 out 6 AD patients and 6 out of 6 controls. The cause of death in all AD and control patients was either respiratory failure or cardiac failure. All tissues were paraformaldehyde (PFA)-fixed, paraffin-embedded and cut to 10 μm thick slices. Sections were deparaffinized with xylene and rehydrated to distilled water after serial ethanol washes. Subsequently, heat-induced antigen retrieval (HIAR) was performed following Dako’s protocol. The tissue sections were then blocked in 5% donkey serum (Jackson ImmunoResearch, West Grove, PA, USA) containing 0.3% Triton X-100 (Sigma-Aldrich, St. Louis, MO, USA), and then incubated with the following primary antibodies overnight at 4 °C: goat anti-human PDGFRβ (1:100, R&D Systems, Minneapolis, MN, USA), mouse anti-human aminopeptidase N (CD13) (1:100, R&D Systems), rabbit anti-human Aβ (1:100, Cell signaling, Boston, MA, USA). Blood vessels were stained by DyLight 488 Labeled *Lycopersicon esculentum* (Tomato) Lectin (1:100, Vector Laboratories, Burlingame, CA, USA, # DL-1174) for 1 h at room temperature. Species-specific fluorochrome-conjugated secondary antibodies were incubated for 1 h at room temperature, including Alexa 568-conjugated donkey anti-goat (1:200, Invitrogen), Alexa 568-conjugated donkey anti-mouse (1:200, Invitrogen) and Alexa 647-conjugated donkey anti-rabbit (1:200, Invitrogen). Tissue sections were mounted and coverslipped using fluorescent mounting media (Dako, Carpinteria, CA, USA). All slices were scanned using Zeiss 510 confocal microscopy with Zeiss Apochromat water immersion objectives (Carl Zeiss MicroImaging Inc., Thornwood, NY, USA). All slices were scanned using Zeiss 510 confocal microscopy with Zeiss Apochromat water immersion objectives (Carl Zeiss MicroImaging Inc., Thornwood, NY, USA), with lasers and band-pass filter settings as the following: a 488-nm argon laser to excite Alexa Fluor and Dylight 488, and the emission was collected through a 500–550-nm bp filter; a 543 HeNe laser to excite Alexa Fluor 568 and Cy3 and the emission was collected through a 560–615-nm bp filter; a 633 HeNe laser to excite Alexa fluor 647 and the emission was collected through a 650–700-nm bp filter.

### Aβ uptake on freshly isolated mouse brain cortical slices

Brain slices were prepared from 2-month old C57BL6 mice or *Lrp*^*lox/lox*^*;Cspg4-Cre* mice. Following the urethane anesthesia, brains were quickly removed from the cranial cavity and sectioned with McIlwain tissue chopper (Ted Pella, Inc., Redding, CA, USA) into 200 μm thick slices containing cortex and hippocampus, as previously described [48]. Slices were recovered for 30 min in a submersion chamber filled with the per-warmed (at 37 °C) artificial cerebrospinal fluid (aCSF; 126 mM NaCl, 2.5 mM KCl, 1.25 mM Na_2_PO_4_, 26 mM NaHCO_3_, 1 mM MgCl_2_, 2 mM CaCl_2_, 0.5 mM ascorbic acid, 2 mM sodium pyrurate, and 10 mM glucose, saturated with 95% O_2_ and 5% CO_2_). Prior to incubation with aCSF containing 5 μg/ml Cy3-Aβ42 for 30 min, slices were pre-incubated with either non-immune IgG (NI-IgG) or LRP1-specific blocking antibody [22, 29] (anti-LRP1 N20, 50 μg/ml; Santa Cruz Biotech., Santa Cruz, CA, USA) for 20 min at 37 °C. At the end of the experiment, brain slices were washed with phosphate buffer saline (PBS) and fixed with 4% PFA for 30 min. The same immunostaining procedure as for human brain tissue without antigen retrieval was performed on mouse brain slices. Primary antibodies included goat anti-mouse aminopeptidase N (CD13) (1:100, R&D Systems), and goat anti-mouse PDGFRβ (1:100, R&D Systems). Secondary antibodies were: Alexa 488-conjugated donkey anti-goat (1:200, Invitrogen). Images were obtained with Zeiss 510 confocal microscopy (Carl Zeiss MicroImaging Inc.) with lasers and band-pass filter settings as described above. The distribution of Cy3-Aβ42 in pericytes and capillary endothelium was analyzed with the NIH Image J software [49].

### Pericyte isolation from the mouse brain, culture and transfection

Brain capillaries were isolated from mouse cortices pooled from 3 to 4 mouse brains for each biological replicate. In experiments in Figs. 4 and 5, we used 3–4 independent biological replicates (cultures), as indicated in the legends to these respective figures. Capillaries were isolated as we have described previously [49, 50]. In brief, mouse cortices were macroscopically dissected, and all visible white matter was discarded in ice-cold PBS containing 2% fetal bovine serum (FBS). The brain was homogenized in PBS containing 2% FBS and centrifuged at 6,000 g for 15 min after addition of Dextran (70 kDa, Sigma). The capillary pellet was collected and sequentially filtered through a 45 μm cell strainer (BD Falcon). The remaining pellet on top of the 45 μm cell strainer was collected in PBS and digested for 12 h at 37 °C with collagenase A (Roche, 10103586001), as we previously described. The cells were washed with PBS and then plated in complete medium containing DMEM, 10% FBS, 1% non-essential amino acids, 1% vitamins and 1% antibiotic/antimycotic on plastic (non-coated) tissue culture plates. After 6 to 12 h the non-adherent cells were washed away and fresh medium was replaced every 2–3 days. Cultures were confirmed to be morphological consistent with pericyte cultures and PDGFRβ-positive, SMA-positive, Desmin-positive, GFAP-negative, AQP4-negative, MAP2-negative, NeuN-negative, VWF-negative, and Iba1-negative, as previously described [37, 49]. Transfections were performed with Neon transfection system (Invitrogen, Grand Island, NY, USA) following the manufacturer’s protocol. After optimization, the transfection efficiency with pEGFP plasmid was > 80%. For siRNA transfection, 15 pmol of siRNA in 1 μl was electroporated into 1 × 10^5^ cells in a total volume of 10 μl.

### Clearance of aggregated Cy3-Aβ42 by primary mouse brain pericytes

The multi-spot glass slides (Thermo Scientific, 9991090, Waltham, MA, USA) were coated with Cy3- Aβ42 [29, 51] at 1 μg per spot without cells or with brain capillary pericytes treated with non-immune IgG (NI-IgG) or LRP1-specific blocking antibody [22, 29] (anti-LRP1; 50 μg/ml) or) or apoE-specific blocking antibody (anti-apoE, HJ6.3, 50 μg/ml), scrambled siRNA (si.*Control*) or LRP1 siRNA (si.*Lrp1*) (Dharmacon, E-040764-00-0010) or apoE siRNA (si.*Apoe*) (Dharmacon, E-040885-00-0005), and 500 nM receptor-associated protein (RAP) [52] (EMD Bioscience), lipidated apoE3 (40 nM) or apoE4 (40 nM) [49]. Cells (5,000 per spot) were incubated for 5 days in DMEM containing 10% heat inactivated FBS, penicillin and streptomycin (Invitrogen). Cells were labeled with Cell Tracker Green CMFDA (Invitrogen C7025), and then fixed with 4% paraformaldehyde. Slides were scanned using Zeiss 510 confocal microscopy (Carl Zeiss MicroImaging Inc.). The Cy3-Aβ42 relative intensity was analyzed with the NIH Image J software.

### Purification of apoE from immortalized astrocytes

Lipidated apoE isoform particles were purified from culture media of human apoE3 or apoE4 overexpressing immortalized astrocytes using an affinity column, as we described previously [53]. Briefly, astrocytes were cultured in advanced DMEM (Invitrogen) with 10% FBS. After 90–95% confluency, cells were washed by PBS and further incubated in advanced DMEM with N-2 Supplement (Invitrogen) and 3 mM 25-hydroxycholesterol (Sigma) for 3 days. Collected culture media were applied onto mouse monoclonal antibody against a human apoE (WU E-4) column. Lipidated apoE particles were eluted from the column with 3 M sodium thiocyanate, concentrated using Apollo centrifugal quantitative concentrators (QMWL: 150 kDa, Orbital Biosciences), and dialyzed against PBS.

### Immunoblotting

Cell samples transfected with scrambled siRNA or LRP1 siRNA were washed in cold PBS and lysed with RIPA buffer. Proteins were quantified with BCA protein assay kit (Pierce). An equal amount of protein sample was loaded for SDS-PAGE. We used the following primary antibodies: rabbit anti-mouse LRP1 (1: 20,000, Abcam, Cambridge, MA, USA), β-actin (1:10,000, Sigma), and Aβ antibody (6E10, 1:1000, Covance). Images were scanned using ChemiDoc™ MP imager with LEDs and quantified using Image Lab™ software (Bio-Rad, Hercules, CA, USA).

### Terminal deoxynucleotidyl transferase UTP nick end labeling (TUNEL)

Paraformaldehyde-fixed, paraffin embedded brain tissue sections from *APP*^*Sw/0*^ mice were sectioned at a thickness of 10 μm. Immunofluorescent detection of pericytes (CD13-positive), Aβ and endothelial-specific lectin fluorescence was conducted as described above. The DeadEnd Fluorometric TUNEL system (Promega) was then completed as described by the manufacturer.

### Statistical analysis

All quantified data represent as mean ± s.e.m. Student’s *t*-test or one-way analysis of variance (ANOVA) followed by Bonferroni post-hoc test was used to determine statistically significant differences. A *P* value < 0.05 was considered statistically significant.

## Results

### Aβ accumulation in pericytes from AD patients and APP^Sw/0^ mice

**Fig. 1:**
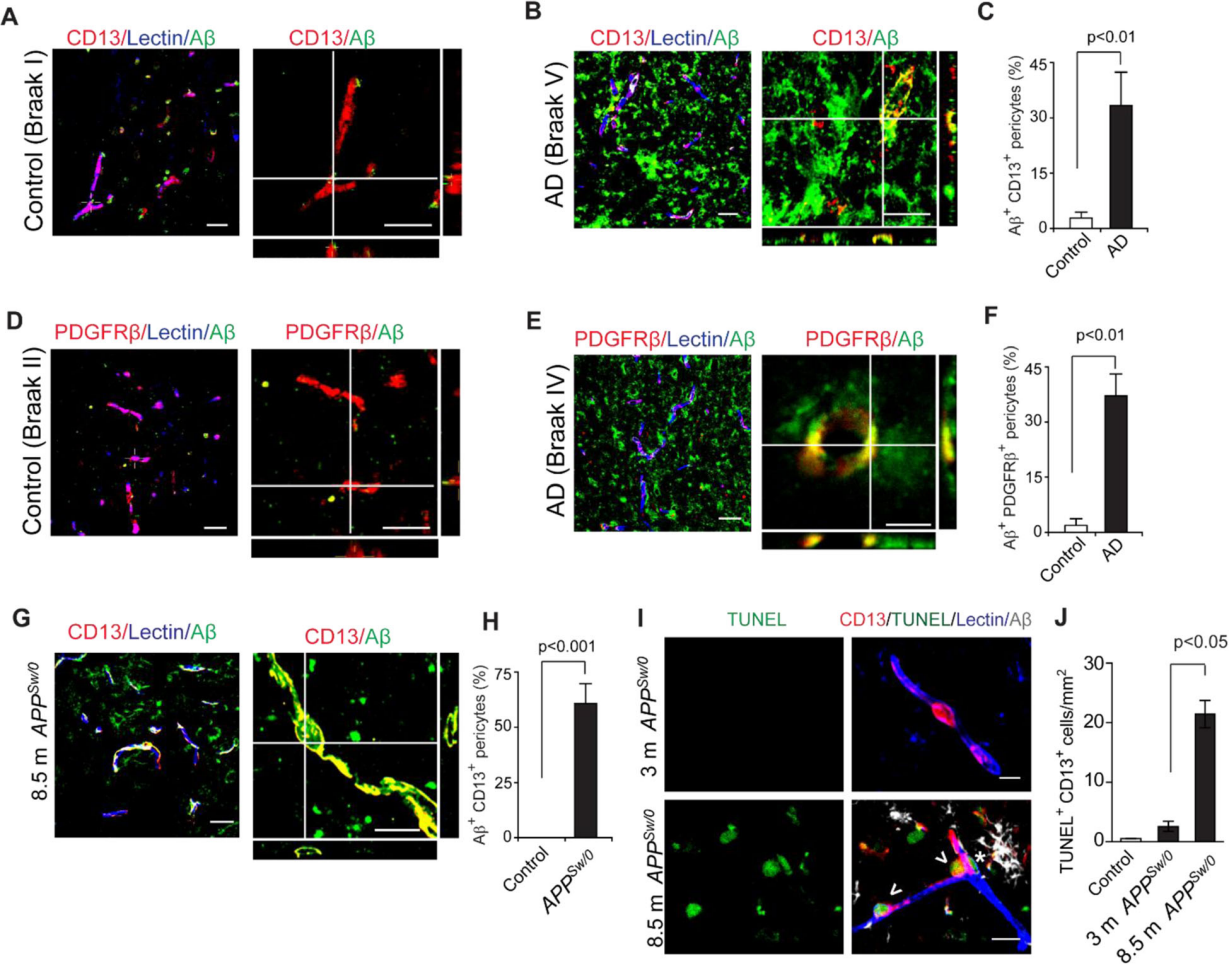
Aβ accumulation in brain pericytes in AD patients and 8.5-month-old *APP*^*Sw/0*^ mice. **a**-**f** Representative confocal microscopy images showing Aβ colocalization with CD13+ (**a**-**b**) and PDGFRβ+ (**d**-**e**) pericytes in brain cortical sections from AD patients compared to negligible levels in age-matched controls, and quantification of Aβ + area in pericytes expressed as the percentage of Aβ + area occupying CD13+ (**c**) or PDGFRβ+ (**f**) pericyte capillary profiles. N = 6 per group; mean ± s.d., *p* < 0.01 by Student’s t-test. Orthogonal views shown on the right in A, B, D and E are from 10 μm Z-stacks. Scale bar, 25 μm. **g**-**i** Representative confocal microscopy images showing Aβ colocalization with CD13+ pericytes (**g**) in brain sections from 8.5-month old *APP*^*Sw/0*^ mice and age-matched littermate control, and quantification of Aβ + area within CD13+ pericyte profiles in *APP*^*Sw/0*^ mice and controls (**h**). N = 3 mice per group; mean ± SD, *p* < 0.001 by Student’s t-test. Orthogonal views from 10 μm Z-stacks. Scale bar, 25 μm. **i** Representative images showing TUNEL staining (green) in CD13+ brain pericytes in 8.5-month old *APP*^*Sw/0*^ mice. Lectin (blue), labels brain endothelium. Aβ (white), shows perivascular and intracellular accumulation. Scale bar: 10 μm. Asterisk shows Aβ deposit; Arrowheads show TUNEL+ CD13+ pericytes. **j** Quantification of TUNEL+ CD13+ pericytes in the cortex of 3- and 8.5-month old *APP*^*Sw/0*^ mice compared to 8.5-month old littermate controls. N = 3 mice per group; mean ± s.e.m.; *p* < 0.05 by Student’s t-test

Using 3-channel confocal microscopy, here we show that Aβ accumulates in > 30% of CD13+ and/or PDGFRβ+ pericytes on lectin+ brain endothelial capillary profiles (< 6 μm in diameter) in brain cortical sections from AD patients compared to barely detectable Aβ accumulation in pericytes in age-matched controls (see demographic Additional file 1: Table S1; Fig. 1a-f). To confirm this observation, we immunostained cortical sections of 8.5-month-old *APP*^*Sw/0*^ mice for human Aβ, at a disease stage when these mice begin depositing Aβ [21]. We found that nearly 60% of CD13+ pericytes were positive for human Aβ (Fig. 1g-h**)**. Collectively, these findings indicate that pericytes in AD and *APP*^*Sw/0*^ mouse brains both accumulate Aβ. Additionally, we found that Aβ also accumulates in brain endothelium of AD patients (Additional file 1: Figure S1A, B), and in lectin+ brain endothelial cells of 8.5-month-old *APP*^*Sw*/0^ mice (Additional file 1:Figure S1C).

### Aβ accumulation is associated with pericyte cell death

Cultured mouse pericytes [37] and human brain pericytes [54] die when treated with excess Aβ. To verify this result in *APP*^*Sw/0*^ mice in vivo, we performed quadruple TUNEL, CD13, lectin and human Aβ staining, which revealed a significant increase in TUNEL+ CD13+ pericytes in 8.5-month old, but not 3-month old, *APP*^*Sw/0*^ mice compared to 8.5-month old littermate controls (Fig. 1i-j), consistent with findings that 3-month old *APP*^*Sw/0*^ mice show barely detectable Aβ deposition in the brain compared to 8.5-month old mice, which begin depositing Aβ at that stage [55].

### LRP1-dependent Aβ uptake by pericytes on mouse brain slices

**Fig. 2:**
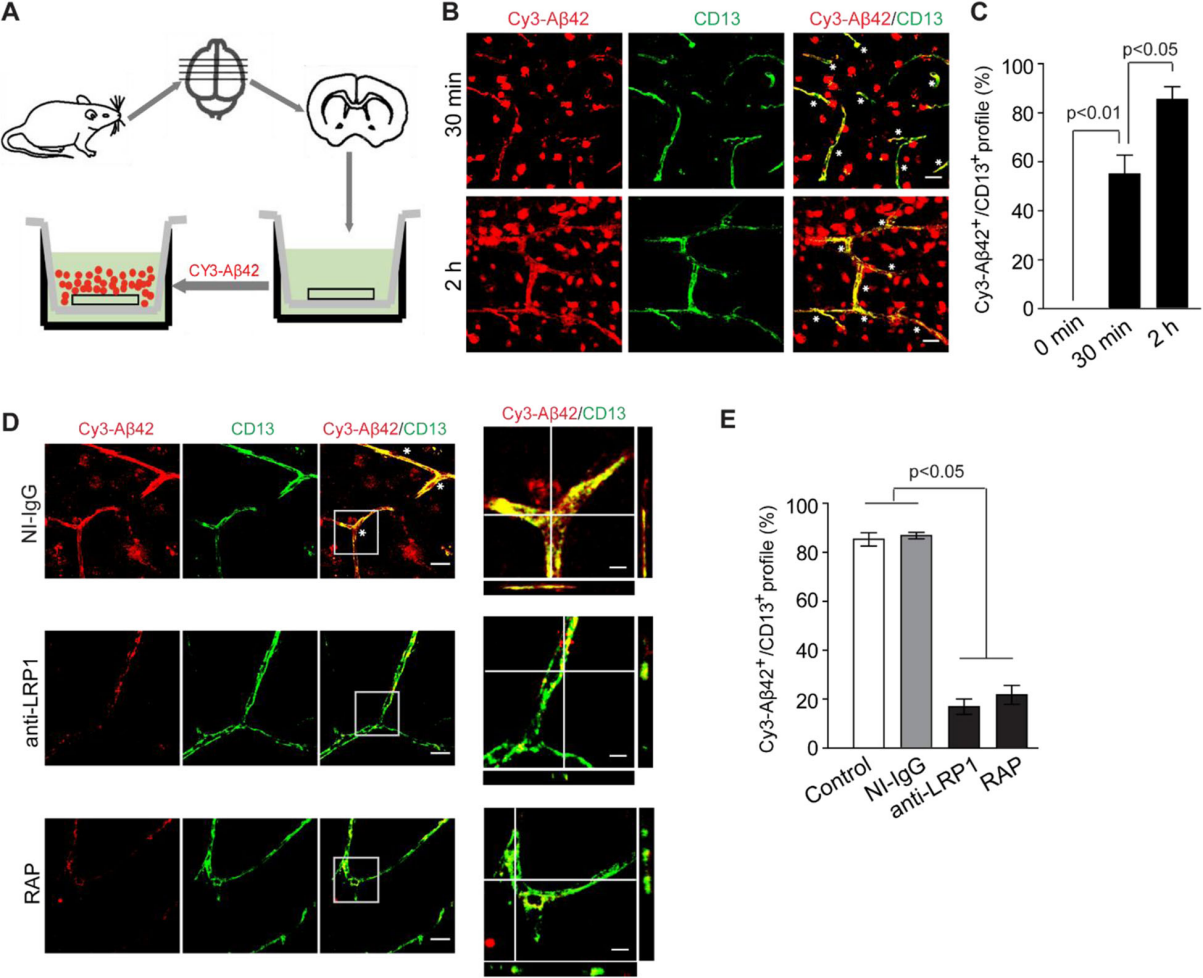
LRP1-dependent Cy3-Aβ42 uptake by pericytes in mouse brain slices. **a** A diagram illustrating the experimental procedure in cultured mouse brain slices used to determine Cy3-Aβ42 uptake by pericytes. Brain slices were first cultured in transwell inserts with oxygenated aCSF (see method) for 4 h before adding Cy3-Aβ42 (1 μM). **b**-**c** Representative low-magnification images (**b**) and quantification (**c**) of cellular uptake of Cy3-Aβ42 by CD13+ pericytes in brain slices at 30 min and 2 h after the addition of Cy3-Aβ42. Scale bar: 25 μm. **d** Representative high magnification images showing Cy3-Aβ42 internalization by CD13-positive pericytes in brain slices in 2 h, in the presence of NI-IgG, anti-LRP1 or RAP. Scale bar: 20 μm. Orthogonal views on the right show Cy3-Aβ42 accumulation in CD13+ pericytes; scale bar: 5 μm. **e** Quantification of Cy3-Aβ42 uptake by CD13+ pericytes in mouse brain slices with and without NI-IgG, anti-LRP1, and RAP. N = 4 independent cultures; mean ± s.e.m.; *p* < 0.05 by One-way ANOVA followed by Bonferroni post-hoc test. Asterisks show colocalization of Cy3-Aβ42 and CD13 signals in (**b**) and (**d**)

To confirm Aβ uptake by pericytes, we next conducted Aβ uptake experiments on freshly isolated acute cortical slice cultures, as previously reported [48]. In this experiment, the mouse brains slices were left to equilibrate in aCSF for 20 min at 37 °C with aeration, that was followed by incubation with Cy3-Aβ42 (1 μM) for 30 min or 2 h. Confocal microscopy was performed to analyze Aβ internalization by pericytes (Fig. 2a). We found that uptake of Cy3-Aβ42 by pericytes was rapid and time-dependent. Compared to 30 min, at 2 h the amount of internalized Cy3-Aβ42 in CD13+ pericytes was further significantly increased (Fig. 2b-c). In these studies, Aβ42 preparation contained a mixture of Aβ42 monomers, dimers, trimers, tetramers, and small molecular weight oligomers (Additional file 1: Figure S2). Both low (Fig. 2b) and high (Fig. 2d) magnification imaging analysis showed strong Cy3-Aβ42 fluorescence signal in CD13+ pericytes and/or PDGFRβ+ pericytes (Additional file 1: Figure S3A-B). In addition to pericytes, Cy3-Aβ42 uptake was observed by endothelial cells, astrocytes and microglia (Additional file 1: Figure S3C-E). The intracellular Cy3-Aβ42 levels in pericytes were significantly reduced in the presence of an anti-LRP1-specific antibody [22, 29] and the receptor associated protein (RAP) [52], an LRP1 antagonist which inhibits ligand binding to LRP1 (Fig. 2d), as shown by 17.1% and 21.7% of the Cy3-Aβ42+ area occupying CD13+ pericyte profiles, respectively, compared to > 80% in control slices treated with vehicle or non-immune immunoglobulin G (IgG) (Fig. 2d-e).

**Fig. 3:**
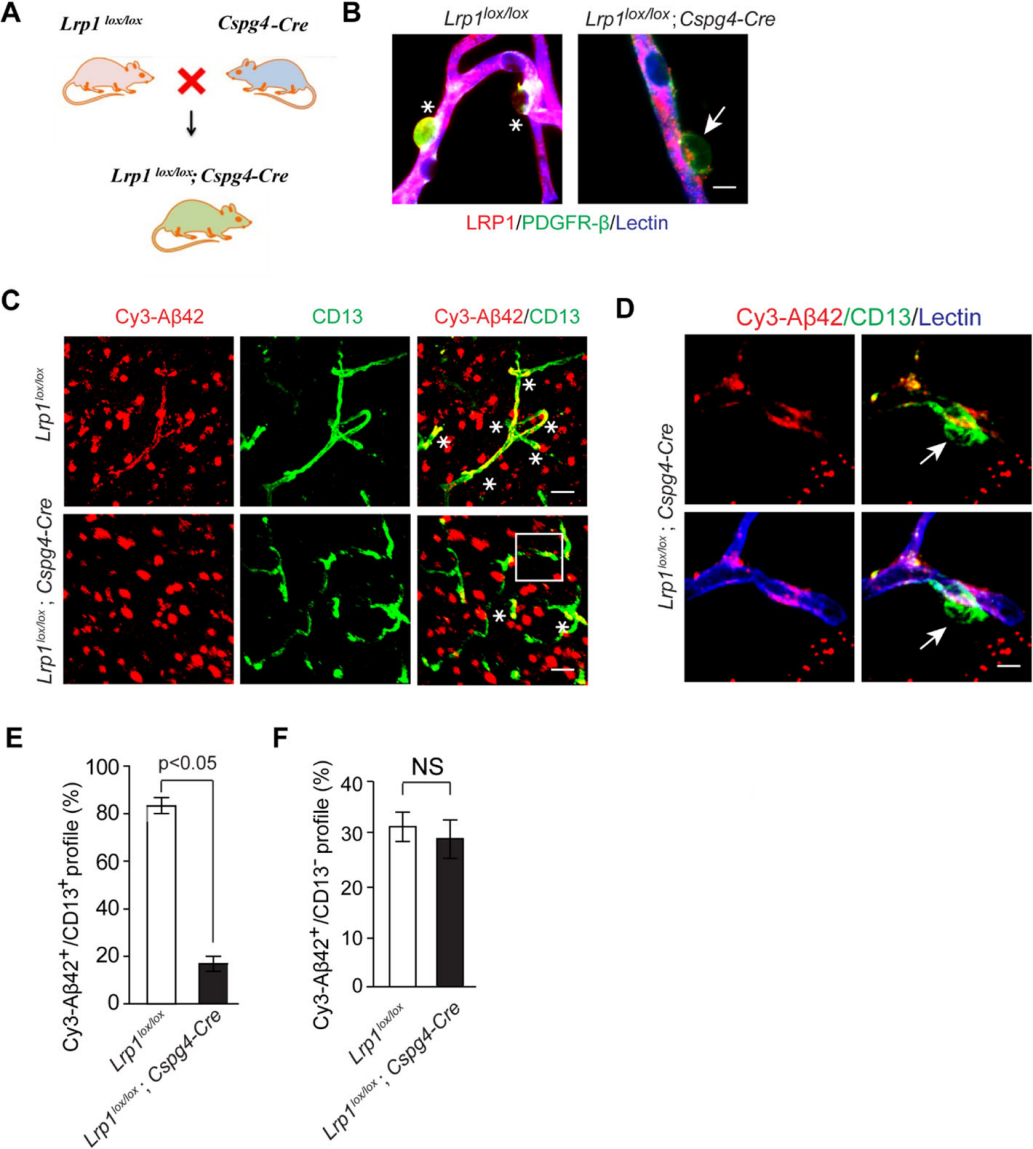
LRP1 genetic deletion from pericytes inhibits Cy3-Aβ42 uptake by pericytes in mouse brain slices. **a** Diagram illustrating generation of *Lrp1*^*lox/lox*^*; Cspg4-Cre* mice by crossing *Lrp1*^*lox/lox*^ mice with *Cspg4-Cre* mice. **b** Representative high magnification images showing LRP1 expression in CD13+ pericytes on isolated murine brain capillaries from control *Lrp1*^*lox/lox*^ mice, but not *Lrp1*^*lox/lox*^*; Cspg4-Cre* mice with LRP1 deletion from pericytes. Asterisks show LRP1 immunostaining in CD13+ pericytes in control *Lrp1*^*lox/lox*^ mice; arrow shows loss of LRP1 immunoreactivity in *Lrp1*^*lox/lox*^*; Cspg4-Cre* mice. Scale bar: 5 μm. **c** Representative images showing Cy3-Aβ42 internalization by CD13+ pericytes in brain slices from control *Lrp1*^*lox/lox*^ mice, and a substantial loss of Cy3-Aβ42 uptake by pericytes in *Lrp1*^*lox/lox*^*; Cspg4-Cre* mice. Asterisks show colocalization between Cy3-Aβ42 and CD13 signals. Scale bar: 25 μm. **d** High magnification images showing greatly reduced Cy3-Aβ42 internalization by a CD13+ pericyte on brain slices from *Lrp1*^*lox/lox*^*; Cspg4-Cre* mice, in contrast to Aβ uptake by lectin-positive endothelial cells. Arrow points to pericyte lacking Cy3-Aβ42 signal. Scale bar: 5 μm. **e**, **f** Quantification of Cy3-Aβ42 cellular uptake by CD13+ pericytes (**e**) compared to all other CD13- (negative) brain cells (**f**) in brain slices from control *Lrp1* ^*lox/lox*^ and *Lrp1* ^*lox/lox*^*; Cspg4-Cre* mice. N = 3 mice per group; mean ± s.e.m.; NS, not significant, p < 0.05 by Student’s t-test

To further investigate the role of LRP1 in Cy3-Aβ42 uptake by pericytes, we generated conditional knockout mice with LRP1 deficiency in pericytes (*Lrp1*^*lox/lox*^*; Cspg4-Cre*) by crossing *Lrp1*^*lox/lox*^ mice [44, 45] with *Cspg4-Cre* mice [46] (Fig. 3a). As expected, CD13+ pericytes on isolated brain microvessels from *Lrp1*^*lox/lox*^*; Cspg4-Cre* mice did not show a detectable LRP1 immunoreactivity in contrast to pericytes from control *Lrp1*^*lox/lox*^ mice, whereas LRP1 remained expressed in lectin+ endothelium in both *Lrp1*^*lox/lox*^*; Cspg4-Cre* and *Lrp1*^*lox/lox*^ microvessels (Fig. 3b), confirming selective deletion of LRP1 from brain capillary pericytes. Both, *Lrp1*^*lox/lox*^ and *Lrp1*^*lox/lox*^*; Cspg4-Cre* mice had comparable CD13+ pericyte coverage (Additional file 1: Figure S4). Next, we studied Cy3-Aβ42 uptake on brain slices from these mice, as described above. Two hours after incubation, Cy3-Aβ42 uptake by CD13+ pericytes was reduced by > 80% in *Lrp1* ^*lox/lox*^*; Cspg4-Cre* mice compared to control *Lrp1* ^*lox/lox*^ mice (Fig. 3c-e). As expected, Aβ uptake by other cell types shown collectively as CD13- cells was not affected in this *Lrp1* conditional knockout model (Fig. 3f). In summary, these data demonstrate LRP1-depedent uptake of Aβ42 species by pericytes.

### LRP1/apoE-dependent clearance of aggregated Cy3-Aβ42 by mouse brain capillary pericytes

**Fig. 4:**
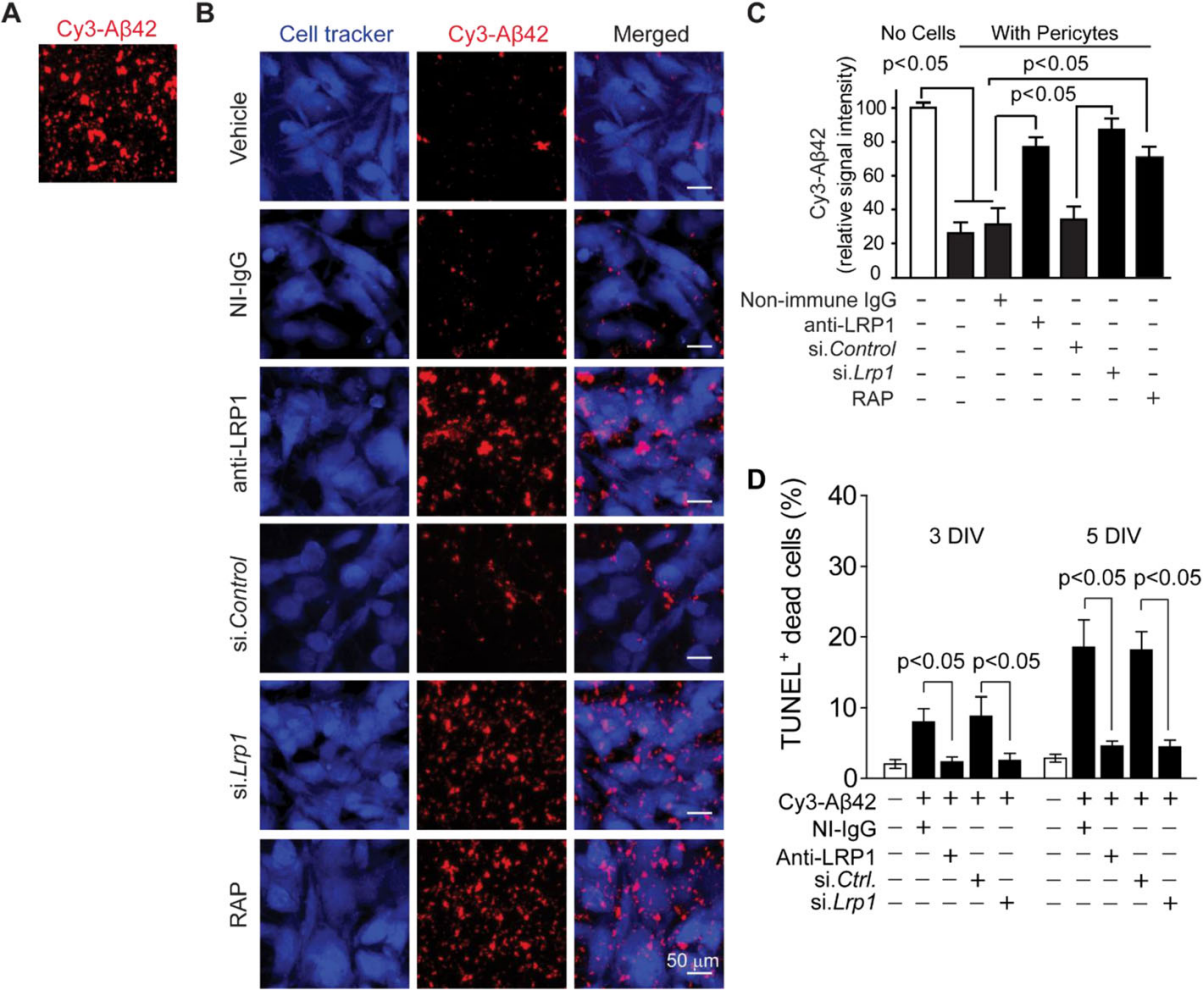
LRP1 mediates clearance of aggregated Cy3-Aβ42 by mouse pericytes. **a**-**b** Multiphoton/confocal laser scanning microscopy of multi-spot glass slides coated with Cy3-Aβ42 without cells (**a**), and with primary mouse brain pericytes cultured for 5 days in the presence of NI-IgG or anti-LRP1, after si.*Lrp1* silencing compared to scrambled si.*Control*, and with RAP or vehicle (**b**). Scale bar, 50 μm. **c** Quantification of Cy3-Aβ42 relative signal intensity on multi-spot slides after 5 days without cells (open bar on the left) and with pericytes in the presence of vehicle (control), NI-IgG and anti-LRP1, after silencing with scrambled si.*Control* or si.*Lrp1*, and in the presence of RAP. N = 4 independent cultures (biological replicates, see Methods); mean ± s.e.m.; p < 0.05 by One-way ANOVA followed by Bonferroni post-hoc test. **d** Quantification of TUNEL+ pericyte cell death at 3 and 7 days after seeding on multi-spot glass slides coated with Cy3-Aβ42 in the presence and absence of NI-IgG and anti-LRP1, and after si.*Lrp1* silencing or si.*Ctrl* as in (**b**). N = 3 independent cultures per group; mean ± s.e.m.; p < 0.05 by One-way ANOVA followed by Bonferroni post-hoc test

To determine whether pericytes can clear aggregated Aβ, we used a slightly modified Aβ clearance model with pericytes seeded on multi-spot glass slides pre-coated with aggregated Cy3-Aβ42, as we reported previously in studies with primary VSMCs [29] (Fig. 4a). Five days after culture, the majority of Aβ42 aggregates (> 70%) were removed from the multi-spot surface by pericytes in the presence of vehicle, control non-immune IgG or scrambled short interfering si.*Control* (Fig. 4b-c), when compared to cell-free control (Fig. 4a). In contrast, only 22.8%, 27.3% and 12.9% of Cy3-Aβ42 was cleared in the presence of anti-LRP1 [22, 29], RAP [52] and *si.Lrp1* (Fig. 4c), respectively. In silencing experiment, *si.Lrp1* efficiently downregulated LRP1 in pericytes by ~ 90% when compared to si.*Control* (Additional file 1: Figure S5A). We also found a time-dependent increase in cell death of pericytes cultured on Aβ pre-coated slides, from 8.2% at 3 DIV to 18.7% at 5 DIV, whereas inhibiting LRP1 by an anti-LRP1 antibody or LRP1 silencing (si.*Lrp1*), not only significantly reduced Aβ uptake, but also diminished pericyte cell death by approximately 3 and 4-fold at 3 and 5 DIV, respectively (Fig. 4d), suggesting that reducing Aβ uptake decreases Aβ toxicity consistent with previous studies with soluble Aβ [37] or Dutch Aβ peptides [54].

**Fig. 5:**
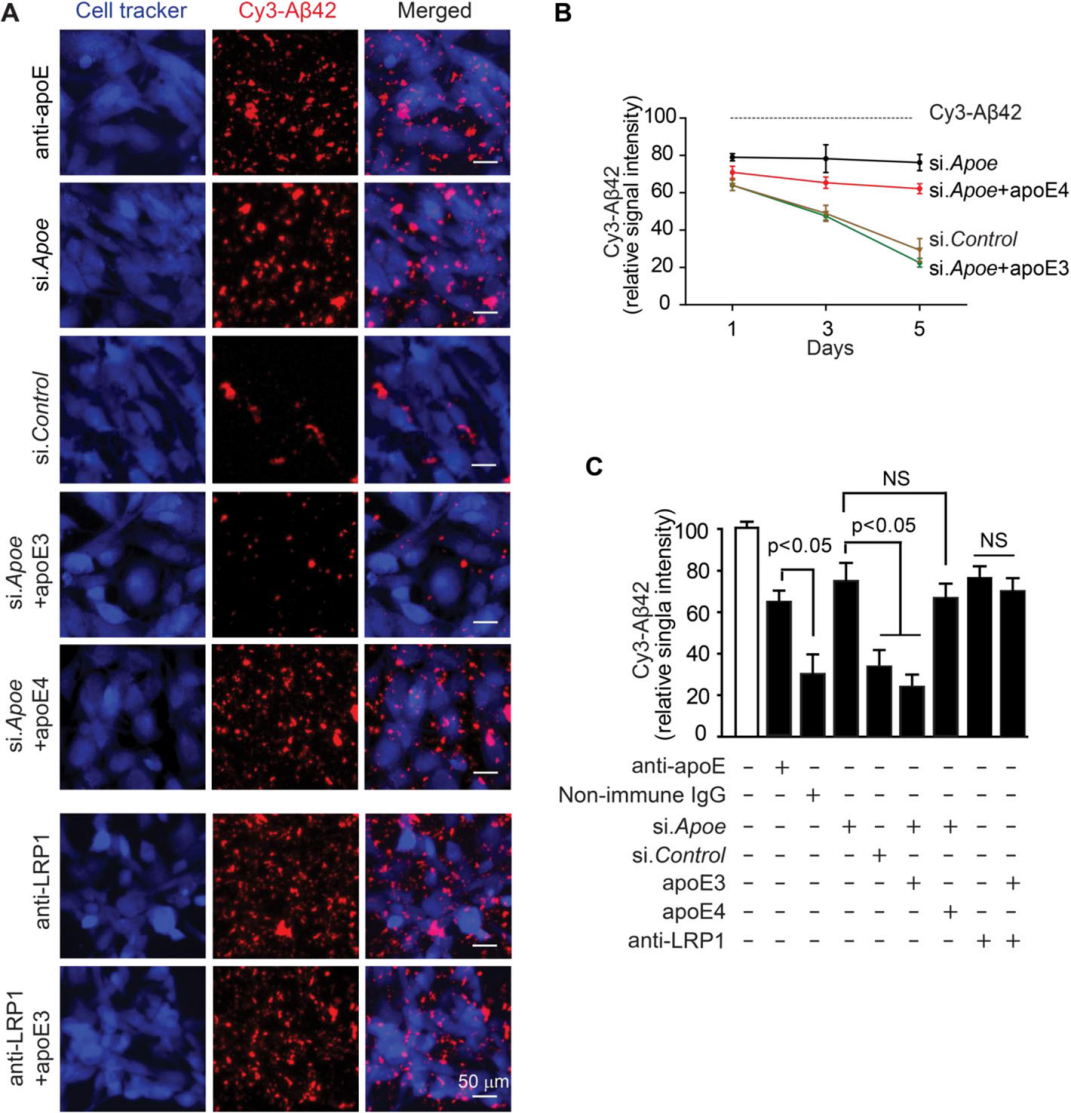
Apolipoprotein E-dependent and isoform-specific effect on LRP1-mediated clearance of aggregated Cy3-Aβ42 by mouse pericytes. **a** Multiphoton/confocal laser scanning microscopy of multi-spot glass slides coated with Cy3-Aβ42 with primary mouse brain pericytes cultured for 5 days in the presence of mouse apoE-specific blocking antibody (anti-apoE), after silencing mouse endogenous apoE (si.*Apoe*) compared to scrambled si.*Control*, and after silencing mouse apoE (si.*Apoe*) in the presence of astrocyte-derived lipidated human apoE3 or apoE4 (40 nM) with and without anti-LRP1 antibody. Scale bar, 50 μm. **b** Time-dependent Cy3-Aβ42 clearance by mouse pericytes cultured for 1, 3 and 5 days after silencing mouse endogenous apoE (si.*Apoe*) compared to si.*Control*, and in the presence of human apoE3 or apoE4. Dashed line indicates Cy3-Aβ42 signal in the absence of cells (control without cells). **c** Quantification analysis of Cy3-Aβ42 relative signal intensity on multi-spot slides after 5 days of culture with pericytes under different experimental conditions as indicated. Gray bar shows Cy3-Aβ42 signal in the absence of cells (control without cells). N = 3 independent cultures; mean ± s.e.m.; p < 0.05 by one-way ANOVA followed by Bonferroni post-hoc test

To check whether apoE is required for LRP1-mediated clearance of Aβ aggregates, we studied clearance by pericytes in the presence of an apoE specific blocking antibody compared to non-immune IgG, and after silencing mouse apoE (si.*Apoe*) compared to scrambled si.*Control* (Additional file 1: Figure S5B; Fig. 5a). After 5 days, we found substantially reduced Aβ clearance with either pharmacologic or genetic inhibition of murine apoE in pericytes as illustrated by representative confocal microscopy images (Fig. 5a), quantification of time-dependent Cy3-Aβ42 clearance by mouse pericytes cultured for 1, 3 and 5 days after silencing mouse endogenous apoE (si.*Apoe*) compared to si.*Control* (Fig. 5b), and quantification analysis of Cy3-Aβ42 relative signal intensity on multi-spot slides after 5 days of culture with pericytes under different experimental conditions (Fig. 5c). These data suggest that pericyte-derived apoE is required for clearance of Aβ aggregates by cultured pericytes, which we show is mediated by LRP1, an apoE receptor (see Fig. 2d-e; Fig. 3c-e and Fig. 4b-c). Besides the self-autonomous effect of apoE in regulating clearance of Aβ aggregates by pericytes (Fig. 5a-c), to mimic in vivo situation we next studied the non-autonomous effects of astrocyte-derived apoE by adding lipidated human apoE3 or apoE4 particles prepared from immortalized astrocytes, as previously described [53]. Previous work has shown that astrocyte-derived apoE provides a major source of apoE in the brain in vivo and signals pericytes via LRP1, but not LRP2, very low density lipoprotein receptor (VLDLR), low density lipoprotein receptor (LDLR) or apoER2 [49]. The effects of apoE3 and apoE4 on Aβ clearance by pericytes was studied after silencing mouse apoE (si.*Apoe*). ApoE3, but not apoE4, almost completely reversed Aβ clearance by pericytes with inhibited mouse apoE (Fig. 5a-c), suggesting that astrocyte-derived apoE exerts a non-autonomous isoform-specific effect on Aβ clearance by pericytes. To additionally confirm that apoE3 effect on pericytes requires LRP1 as previously shown [49], we performed the same experiment in the presence of an anti-LRP1 antibody. As expected, this experiment showed that anti-LRP1 inhibits apoE3’s ability to reverse the Aβ clearing capability of mouse pericytes with silenced mouse apoE (si.*Apoe*) (Fig. 5a, c).

## Discussion

Our data show that BBB-associated pericytes accumulate an abundance of Aβ on brain capillaries in AD patients and *APP*^*Sw/0*^ mice. We also show that pericytes play a major role in clearance of different Aβ42 species including a mixture of monomers and small molecular weight oligomers, which is mediated via LRP1, similar as previously shown for Aβ40 [37]. This has been demonstrated by pharmacologic inhibition of LRP1 by an anti-LRP1 antibody [22, 29] and RAP [52], genetic *Lrp1* knockdown with short interfering RNA (si.*Lrp1*) and deletion of LRP1 from pericytes in *Lrp*^*lox/lox*^*;Cspg4-Cre* mice compared to *Lrp*^*lox/lox*^ controls. Moreover, we show that pericytes efficiently clear Aβ42 aggregates by an LRP1/apoE isoform-specific mechanism. This has been demonstrated by silencing mouse *Apoe* (si.*Apoe*) in pericytes in the absence and presence of human astrocyte-derived lipidated apoE3 or apoE4, which revealed that apoE3, but not apoE4, mediates LRP1-dependent clearance of Cy3-Aβ42 aggregates. Overall, these data point to Aβ clearance on pericytes as a possible contributory factor in the pathogenesis of AD and accumulation of Aβ in the brain and around brain capillaries causing capillary CAA as seen in AD [8]. The data also suggest that LRP1/apoE interaction on pericytes should be explored further as a potential therapeutic approach for controlling Aβ clearance in AD.

Accumulation of Aβ in pericytes in AD and *APP* mouse brain capillaries likely reflects Aβ overload that exceeds pericytes clearance capability resulting in intracellular trapping of Aβ. This in turn may set a stage for the formation of amyloid deposits around brain capillaries and within the basement membrane between pericytes and endothelial cells, as shown in AD [7, 8] and *APP* models [21]. Consistent with these findings we also observed accumulation of Aβ in the brain endothelium in both AD and *APP*^*Sw/0*^ mice likely reflecting excess Aβ that has not been cleared via transport across the BBB [27]. Collectively, these data suggest that not only previously shown faulty clearance of Aβ on VSMCs contributes to CAA and Aβ pathology [29, 30], but also impaired clearance on pericytes may contribute to development of capillary CAA [8, 21] and retention of Aβ in the brain. This might be particularly important at disease stages when other clearance mechanisms including Aβ drainage by the perivascular route and/or the meningeal lymphatics become deficient, as recently shown in *APP* mouse models [16, 17].

Pericyte degeneration and loss have been reported in AD patients [47, 56–59] and *APP* mice [37, 60]. Consistent with previous findings demonstrating that excess Aβ in pericytes can trigger cell death in human [54] and mouse [37] pericytes, we also found that pericytes in *APP*^*Sw/0*^ mice die at the time of Aβ deposition in the brain and its accumulation in pericytes. Pericyte cell death, in turn, can exacerbate progression of AD pathology, as shown in *APP*^*Sw/0*^; *Pdgfrb*^*+/−*^ mice with accelerated pericyte loss [37]. Similar as shown in human pericytes [54], we also found that LRP1 mediates both Aβ internalization and cell death of mouse pericytes, as illustrated by diminished Aβ uptake and reduced cell death in the presence of LRP1 inhibition by either an anti-LRP1 antibody and/or *Lrp1* silencing. Consistent with the present findings, previous studies by Verbeek and colleagues [54, 61–63] suggested that Aβ effects on pericytes are modulated by apoE isoforms. In brief, they showed that human pericytes from apoE4 carriers compared to apoE3 carriers secrete less apoE in the culture media resulting in increased accumulation of Dutch Aβ peptide on the cell surface and greater rate of cell death induced by Aβ [63].

Interestingly, loss of pericytes in human AD is significantly higher in apoE4 carriers compared to apoE3 carriers, which is associated with greater degree of BBB breakdown [56, 64], and increased risk for CAA, as shown both in human apoE4 compared to apoE3 carriers [65–69], and *APP* mouse models on apoE4 compared to apoE3 background [66, 70]. These findings are consistent with the present data showing that human astrocyte-derived apoE4, in contrast to apoE3, cannot reverse LRP-1-mediated Aβ clearance by mouse pericytes with silenced mouse endogenous apoE, and previous findings demonstrating that apoE4 compared to apoE3 poorly binds to LRP1, which leads from on one hand to BBB breakdown by activating BBB-degrading cyclophilin-A-matrix metallopropteinase-9 pathway in pericytes [49], and from the other, diminished clearance of Aβ across the BBB [39].

Finally, the present findings showing that pericytes actively contribute to Aβ removal at the BBB via LRP1-mediated apoE isoform-dependent clearance on brain capillaries should encourage future studies directed at exploring possible therapeutic potential of this pathway to control CAA and Aβ pathology in AD. For example, pharmacologic or genetic strategies that can increase activity of LRP1/apoE clearance system in pericytes and at the BBB might diminish CAA and Aβ accumulation in apoE3 carriers, but may not work as well in apoE4 carriers. On the other hand, recent cell therapy studies have shown that mouse mesodermal pericytes can improve cerebral blood flow and reduce Aβ pathology when injected into the brain of *APP* mice [38]. Based on the present findings, this therapeutic effect likely depends on mouse endogenous apoE. When translating this approach to humans, based on the present findings one can envisage using iPSC-derived pericytes from apoE3 carriers as a straight Aβ lowering cell clearance therapy, whereas in case of apoE4 carriers, CRISPER/Cas9 approach could be used to generate apoE3-secreting iPSC-derived pericytes with enhanced Aβ clearance properties.

## Conclusions

In conclusion, our findings show that BBB-associated pericytes clear Aβ aggregates via an LRP1-dependent apoE isoform-specific mechanism with apoE4 disrupting Aβ clearance compared to apoE3. Overall, the present data support that the LRP1/apoE pathway in pericytes has a potential to be explored as a therapeutic target for controlling Aβ clearance and levels in AD.

## Additional file

Additional file 1: **Table S1.** Demographic and clinical features of human subjects used in this study. **Figure S1.** Aβ deposition in microvessels in AD patients and *APP*^Sw/0^ mice. **Figure S2.** Biochemical analysis of Aβ42 aggregates. **Figure S3.** Cy3-Aβ42 cellular uptake in wild type mouse brain slices within 30 min. **Figure S4.** Pericyte coverages in *Lrp1*^lox/lox^ and *Lrp1*^lox/lox^; Cspg4-Cre mice. **Figure S5.**. LRP1 and apoE suppression with siRNA. (DOCX 1454 kb)

## Abbreviations

aCSF: Artificial cerebrospinal fluid
AD: Alzheimer’s disease
apoE: Apolipoprotein E
*APP*: Aβ-precursor protein
Aβ: Amyloid-β
BBB: Blood-brain barrier
CAA: Cerebral amyloid angiopathy
CD13: Cluster of differentiation 13
CERAD: Consortium to establish a registry for Alzheimer’s disease
Cspg4: Chondroitin sulfate proteoglycan 4
Cy3: Cyanine 3
FBS: Fetal bovine serum
ISF: Interstitial fluid
LRP1: Low density lipoprotein receptor related protein 1
PFA: Paraformaldehyde
PICALM: Phosphatidylinositol binding clathrin assembly protein
RAP: Receptor-associated protein
TUNEL: Terminal deoxynucleotidyl transferase UTP nick end labeling
VSMCs: Vascular smooth muscle cells
