# Correction: Blood-brain barrier-associated pericytes internalize and clear aggregated amyloid-β42 by LRP1-dependent apolipoprotein E isoform-specific mechanism

**DOI:** 10.1186/s13024-022-00573-5

**Published:** 2022-11-03

**Authors:** Qingyi Ma, Zhen Zhao, Abhay P. Sagare, Yingxi Wu, Min Wang, Nelly Chuqui Owens, Philip B. Verghese, Joachim Herz, David M. Holtzman, Berislav V. Zlokovic

**Affiliations:** 1 Center for Neurodegeneration and Regeneration, Zilkha Neurogenetic Institute and Department of Physiology and Neuroscience, Keck School of Medicine, University of Southern California, Los Angeles, California 90033 USA; 2 Lawrence D. Longo, MD Center for Neonatal Biology, Division of Pharmacology, Department of Basic Sciences, Loma Linda University School of Medicine, Loma Linda, CA 92350 USA; 3 C2N Diagnostics, LLC, Saint Louis, MO 63110 USA; 4 Department of Molecular Genetics, University of Texas Southwestern Medical Center, Dallas, TX USA; 5 Department of Neuroscience, University of Texas Southwestern Medical Center, Dallas, TX USA; 6 Department of Neurology and Neurotherapeutics and Center for Translational Neurodegeneration Research, University of Texas Southwestern Medical Center, Dallas, TX USA; 7 Department of Neurology, Hope Center for Neurological Disorders, Knight Alzheimer’s Disease Research Center, Washington University School of Medicine, Saint Louis, MO 63110 USA

**Correction: Mol Neurodegener 13, 57 (2018)**

**https://doi.org/10.1186/s13024-018-0286-0**

After publication of this work [1], an error was noticed in Fig. 4B.

**Fig. 4:**
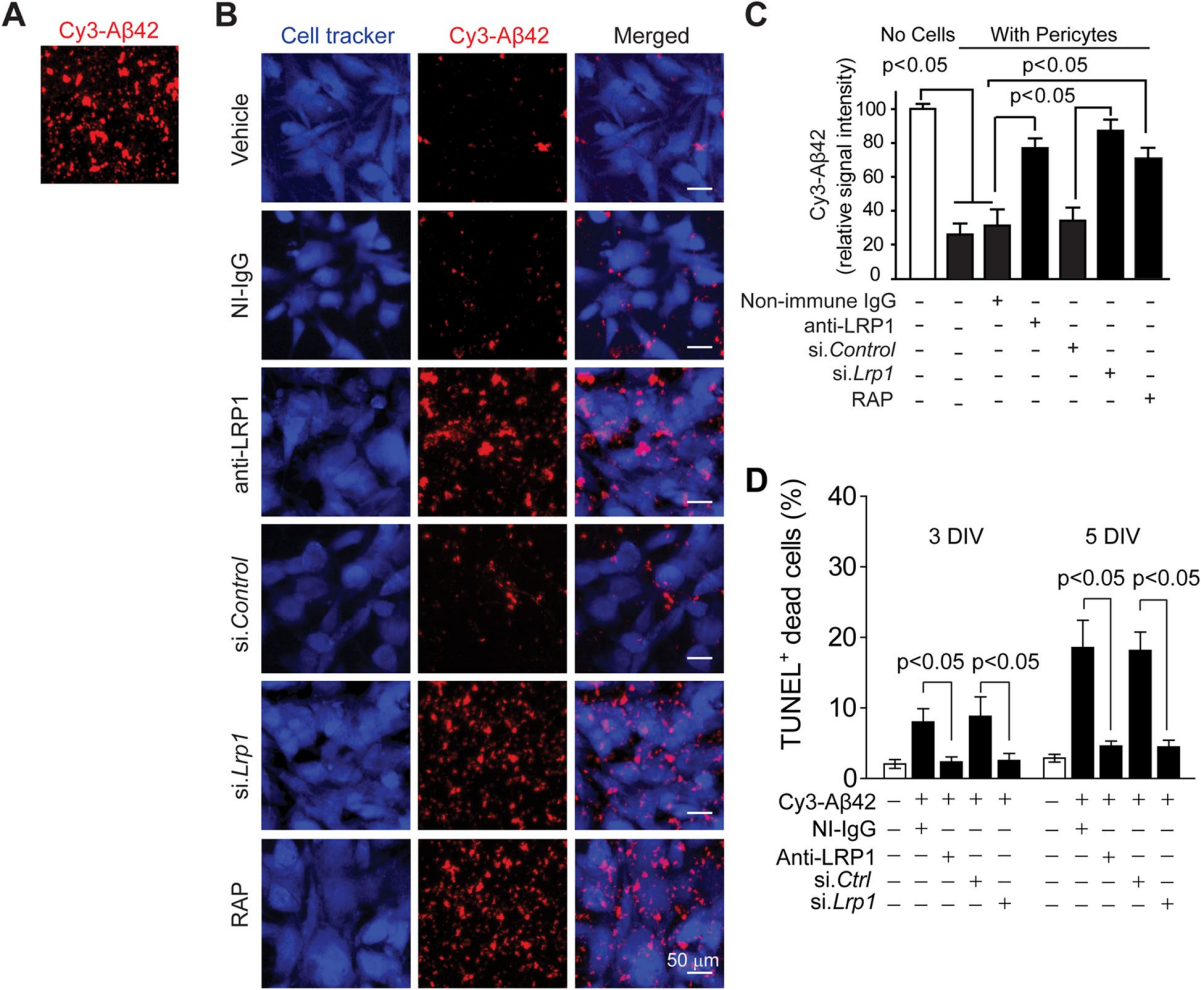
LRP1 mediates clearance of aggregated Cy3-Aβ42 by mouse pericytes. **a**-**b** Multiphoton/confocal laser scanning microscopy of multi-spot glass slides coated with Cy3-Aβ42 without cells (**a**), and with primary mouse brain pericytes cultured for 5 days in the presence of NI-IgG or anti-LRP1, after si.*Lrp1* silencing compared to scrambled si.*Control*, and with RAP or vehicle (**b**). Scale bar, 50 μm. **c** Quantification of Cy3-Aβ42 relative signal intensity on multi-spot slides after 5 days without cells (open bar on the left) and with pericytes in the presence of vehicle (control), NI-IgG and anti-LRP1, after silencing with scrambled si.*Control* or si.*Lrp1*, and in the presence of RAP. N = 4 independent cultures (biological replicates, see Methods); mean ± s.e.m.; *p* < 0.05 by One-way ANOVA followed by Bonferroni post-hoc test. **d** Quantification of TUNEL+ pericyte cell death at 3 and 7 days after seeding on multi-spot glass slides coated with Cy3-Aβ42 in the presence and absence of NI-IgG and anti-LRP1, and after si.*Lrp1* silencing or si.*Ctrl* as in (**b**). N = 3 independent cultures per group; mean ± s.e.m.; *p* < 0.05 by One-way ANOVA followed by Bonferroni post-hoc test

In the Cell Tracker column shown below on the left, an incorrect representative image for Cell Tracker was used for the NI-IgG condition (non-immune IgG control). The error in this representative Cell Tracker image likely occurred when updates were made to the representative images in this figure during preparation of the manuscript by mistake. The Cy3-Aβ image for this NI-IgG condition was correct and does not need to be replaced.

This error only pertains to the incorrect representative Cell Tracker NI-IgG image in Fig. 4B, and does not affect any of the analysis or conclusions presented in the paper, as all quantifications were based on the Cy3-Aβ (red) signal intensity representing Aβ cleared by the cells under different conditions.

After carefully going back through all the raw data, we found and selected the exact correct Cell Tracker image for this particular NI-IgG condition shown in Fig. 4B corresponding to the Cy3-Aβ (red) signal.

We would appreciate to publish erratum and replace incorrect representative Cell Tracker image as shown on the left side below (as published) with the correct representative Cell Tracker image for this particular NI-IgG condition shown below on the right side.

The correct version of the entire Fig. 4 with the correct Cell tracker image for panel 4B NI-IgG representative image is shown below. Nothing else in this figure needs to be changed. The analysis or conclusions presented in the paper remain the same as is, because the quantifications were based on the remaining Cy3-Aβ (red) signal intensity representing Aβ uncleared by the cells.
