# Correction: Blood–brain barrier-associated pericytes internalize and clear aggregated amyloid-β42 by LRP1-dependent apolipoprotein E isoform-specific mechanism

**DOI:** 10.1186/s13024-024-00716-w

**Published:** 2024-03-22

**Authors:** Qingyi Ma, Zhen Zhao, Abhay P. Sagare, Yingxi Wu, Min Wang, Nelly Chuqui Owens, Philip B. Verghese, Joachim Herz, David M. Holtzman, Berislav V. Zlokovic

**Affiliations:** 1 Center for Neurodegeneration and Regeneration, Zilkha Neurogenetic Institute and Department of Physiology and Neuroscience, Keck School of Medicine, University of Southern California, Los Angeles, CA 90033 USA; 2 Lawrence D. Longo, MD Center for Neonatal Biology, Division of Pharmacology, Department of Basic Sciences, Loma Linda University School of Medicine, Loma Linda, CA 92350 USA; 3 C2N Diagnostics, LLC, Saint Louis, MO 63110 USA; 4 Department of Molecular Genetics, University of Texas Southwestern Medical Center, Dallas, TX USA; 5 Department of Neuroscience, University of Texas Southwestern Medical Center, Dallas, TX USA; 6 Department of Neurology and Neurotherapeutics and Center for Translational Neurodegeneration Research, University of Texas Southwestern Medical Center, Dallas, TX USA; 7 Department of Neurology, Hope Center for Neurological Disorders, Knight Alzheimer’s Disease Research Center, Washington University School of Medicine, Saint Louis, MO 63110 USA

**Correction** **:** **Mol Neurodegener 13, 57 (2018)**

**https://doi.org/10.1186/s13024-018-0286-0**

After publication of the first correction [1] to the original manuscript [2] regarding Fig. 4b, errors were noticed in the corrected Fig. 4B representative images for anti-LRP1 and RAP conditions:

- In the merged column, representative images with similar pattern were noticed in anti-LRP1 and si.*Lrp1* conditions, and in the Cy3-Aβ42 column, representative images with similar pattern were noticed in si.*Lrp1* and RAP conditions.
- The anti-LRP1 merged image, an incorrect cell tracker image was used for the merged overlay image. The merged image for anti-LRP1 has been corrected using images from the anti-LRP1 Cell tracker and Cy3-Aβ42 channels as originally presented in Fig. 4B.
- The RAP Cy3-Aβ42 image is incorrect and was also incorrectly used for the RAP merged image. The authors have identified the correct RAP Cy3-Aβ42 image and replaced both the Cy3-Aβ42 and merged RAP images.

The single channel si.*Lrp1* Cy3-Aβ42 image and si.*Lrp1* merged image are both correct, and no change is needed. Importantly, these errors only pertain to the incorrect representative images in Fig. 4B and have no impact on the analysis or conclusions presented in the paper.

The corrected version of the entire Fig. 4 is shown ahead, and the authors apologize for these unintentional errors.

**Fig. 4:**
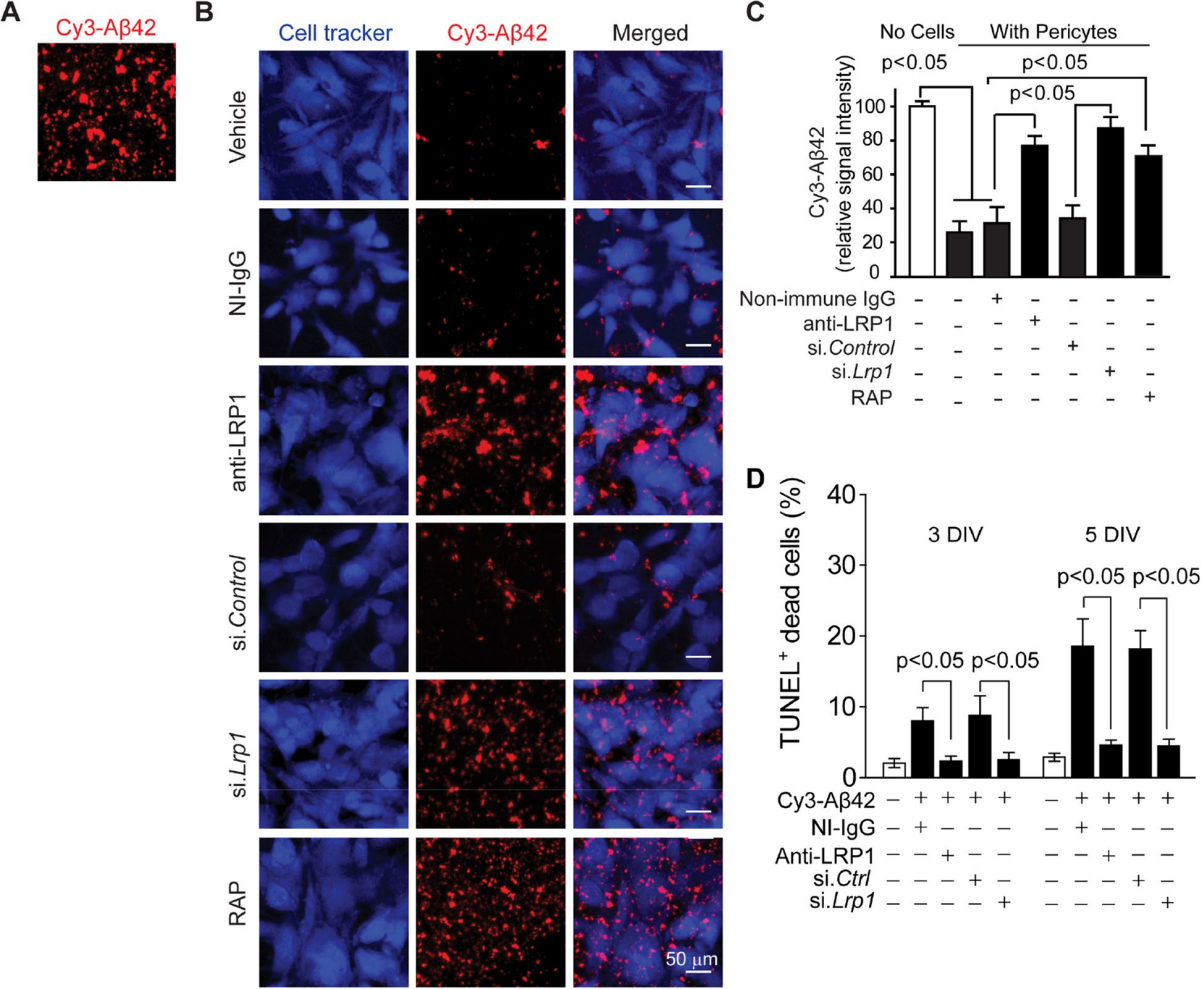
LRP1 mediates clearance of aggregated Cy3-Aβ42 by mouse pericytes. **a**-**b** Multiphoton/confocal laser scanning microscopy of multi-spot glass slides coated with Cy3-Aβ42 without cells (**a**), and with primary mouse brain pericytes cultured for 5 days in the presence of NI-IgG or anti-LRP1, after si.*Lrp1* silencing compared to scrambled si.*Control*, and with RAP or vehicle (**b**). Scale bar, 50 μm. **c** Quantification of Cy3-Aβ42 relative signal intensityon multi-spot slides after 5 days without cells (open bar on the left) and with pericytes in the presence of vehicle (control), NI-IgG and anti-LRP1, after silencing with scrambled si.*Control* or si.*Lrp1*, and in the presence of RAP. *N* = 4 independent cultures (biological replicates, see Methods); mean ± s.e.m.; *p* < 0.05 by One-way ANOVA followed by Bonferroni post-hoc test. d Quantification of TUNEL + pericyte cell death at 3 and 7 days after seeding on multi-spot glass slides coated with Cy3-Aβ42 in the presence and absence of NI-IgG and anti-LRP1, and after si.*Lrp1* silencing or si.*Ctrl* as in (**b**). *N* = 3 independent cultures per group; mean ± s.e.m.; *p* < 0.05 by One-way ANOVA followed by Bonferroni post-hoc test
