# Supplementary material for: Blood-brain barrier-associated pericytes internalize and clear aggregated amyloid-β42 by LRP1-dependent apolipoprotein E isoform-specific mechanism
Source: Mol Neurodegener. 2018 Oct 19;13:57. doi: 10.1186/s13024-018-0286-0 (PMC6194676; doi:10.1186/s13024-018-0286-0)
Supplement: Supplementary file 1 — Table S1. Demographic and clinical features of human subjects used in this study. Figure S1. Aβ deposition in microvessels in AD patients and APPSw/0 mice. Figure S2. Biochemical analysis of Aβ42 aggregates. Figure S3. Cy3-Aβ42 cellular uptake in wild type mouse brain slices within 30 min. Figure S4. Pericyte coverages in Lrp1lox/lox and Lrp1lox/lox; Cspg4-Cre mice. Figure S5.. LRP1 and apoE suppression with siRNA. (DOCX 1454 kb) [file 13024_2018_286_MOESM1_ESM.docx]

**Additional file 1**

**Table S1.**

|  | Controls (n = 6) | AD (n = 6) |
| --- | --- | --- |
| Gender (M/F) | (3/3) | (2/4) |
| Braak stage | 0 (1), 1 (2), II (2), III (1) | III (1), IV (1), V (3), V-VI (3) |
| CERAD | Negative (6/6) | Frequent (6/6) |
| Vascular risk factors | 6/6 | 4/6 |
| *APOE* genotypes | ε3/ε3 (2), ε3/ε4 (4) | ε3/ε3(2), ε3/ε4 (3), ε4/ε4 (1) |
| Age of death | 76 ± 11 | 83 ± 6 |

Demographic and clinical features of human subjects used in this study. Abbreviations:

CERAD = Consortium to Establish a Registry for Alzheimer’s Disease. Mean ± SD.

**Figure S1. Aβ deposition in microvessels in AD patients and *APP ^Swe^* mice**


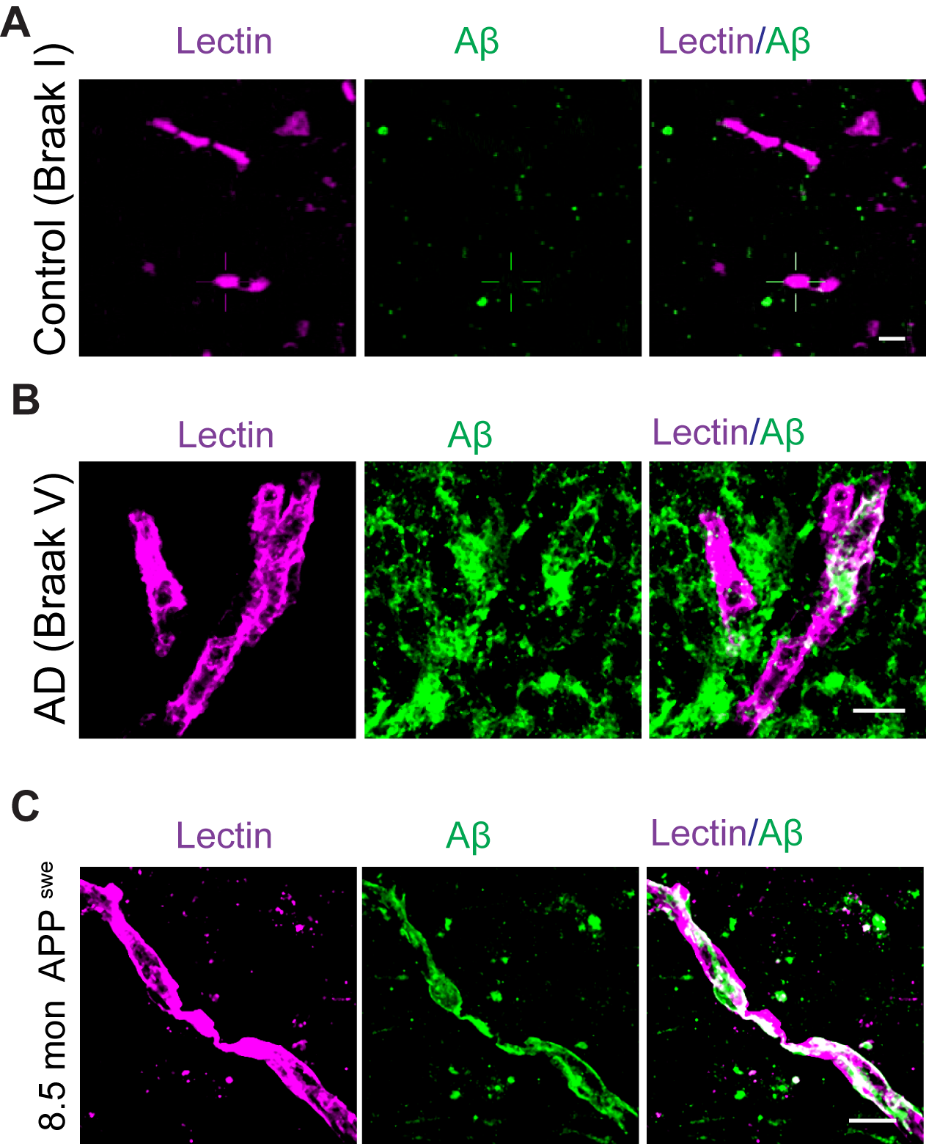


(**A-C**) Representative high resolution confocal microscopy analysis showing Aβ deposition (green) in Lectin-positive brain microvessels (magenta) in human control and AD patient (**A, B**), and 8.5 months old *APP ^sw/0^* mice (**C**). Colocalization is shown as white. Scale bar: 10 µm.

**Figure S2.**

**
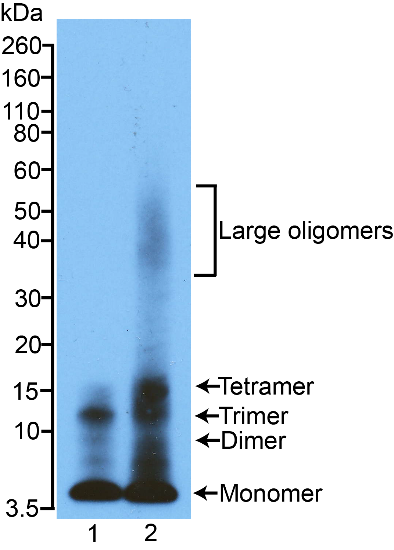
**

Western blot analyses of Aβ42 aggregates separated on a 4-12% Bis-Tris nonreducing SDS-PAGE and probed with the β-amyloid (D54D2) XP rabbit monoclonal antibody (Cat #8243S, Cell Signaling) recognizing the amino terminus of human Aβ. 1 µM Aβ42 in serum-free pericyte culture medium (freshly prepared, lane 1) and after 2 h incubation at 37°C (lane 2).

**Figure S3. Cy3-Aβ42 cellular uptake in wild type mouse brain slices within 30 min.**

**
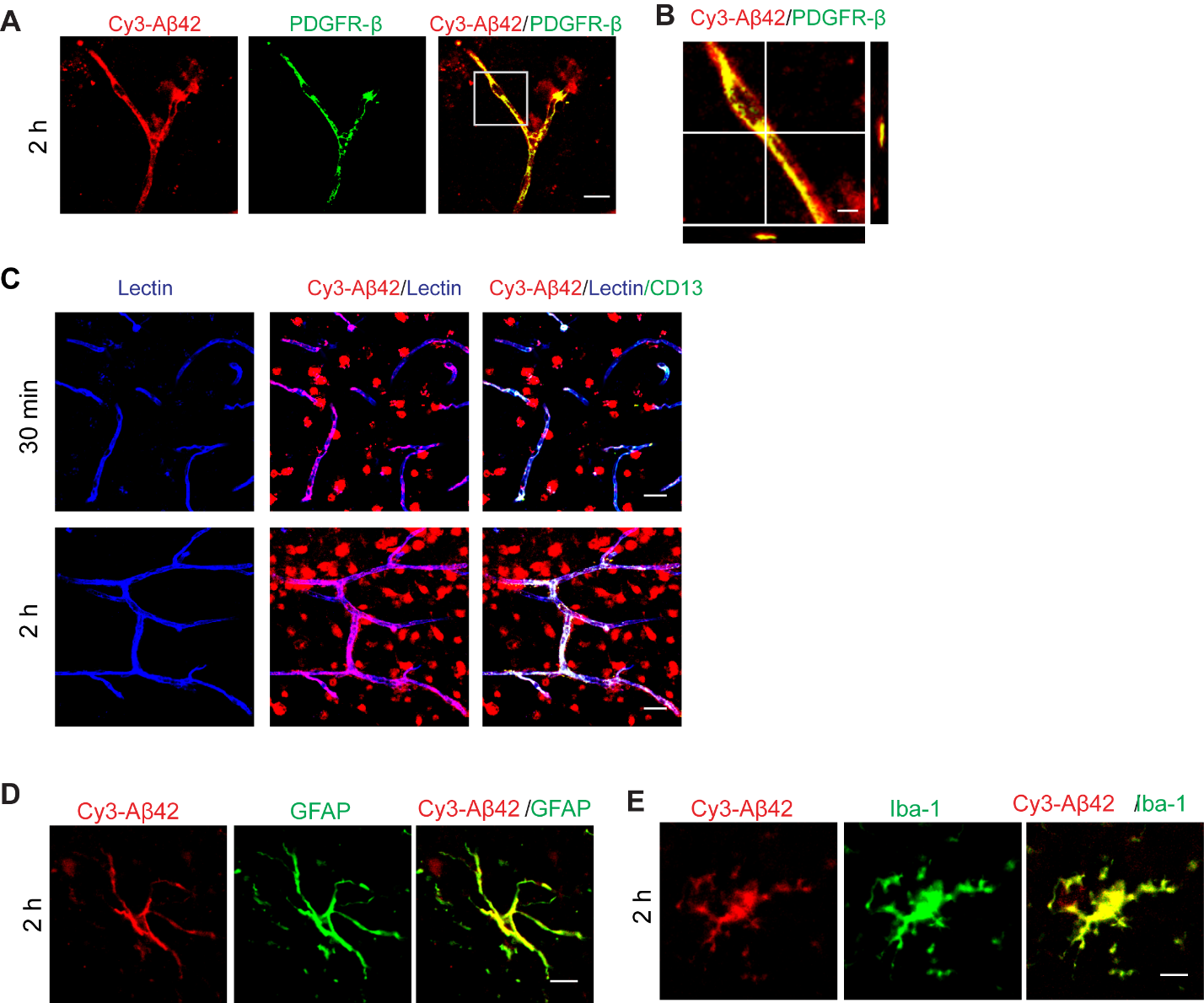
**

**(A)** Representative high resolution confocal microscopy images showing Cy3-Aβ42 (red) uptake by PDGFRβ-positive pericytes (green) in murine brain slices within 30 min in the presence of 5 µg/ml Cy3-Aβ42. Mouse brain slices were prepared as in Fig 5. Scale bar: 5 µm (**B**) Orthogonal views showing Cy3-Aβ42 (red) accumulation inside of a PDGFR-β-positive pericyte (green). Merge: yellow. **(C)** Representative high resolution confocal microscopy images showing Cy3-Aβ42 (red) uptake by lectin-positive endothelial cells (green) in murine brain slices 30 min or 2 h after 5 µg/ml Cy3-Aβ42 treatment. Scale bar: 20 µm. **(D, E)** Representative images showing Cy3-Aβ42 uptake by GFAP-positive astrocyte **(C)** and Iba-1-positive microglia **(D)**. Scale bar: 20 µm.

**Figure S4. Pericyte coverages in *Lrp1^lox/lox^* and *Lrp1^lox/lox^; Cspg4-Cre* mice.**


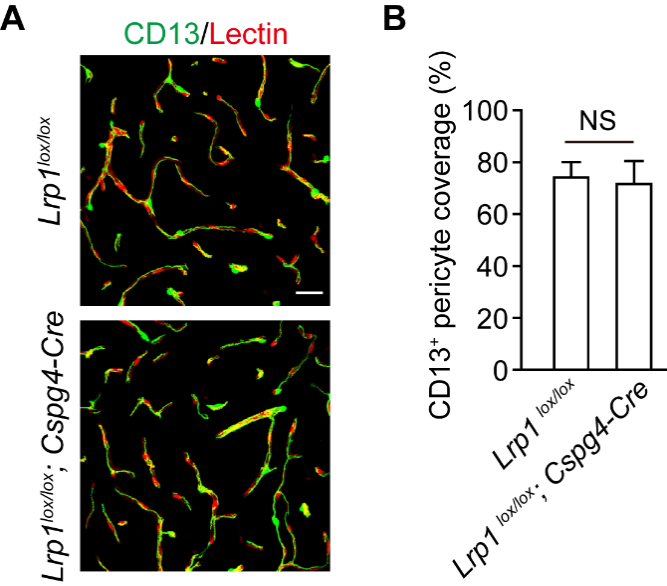


**Figure S4. (A)** Representative confocal images showing CD13-positive pericytes and lectin-positive endothelium in the cortex of *Lrp1^lox/lox^* and *Lrp1^lox/lox^; Cspg4-Cre* mice, as used in Figure 3. Bar = 25 µm. (**B**) Quantification of CD13+ pericyte coverage over lectin+ endothelial profiles. N = 3 mice per group; mean ± s.e.m.; NS, not significant, by Student’s t-test.

**Figure S5. LRP1 and apoE suppression with siRNA**

**
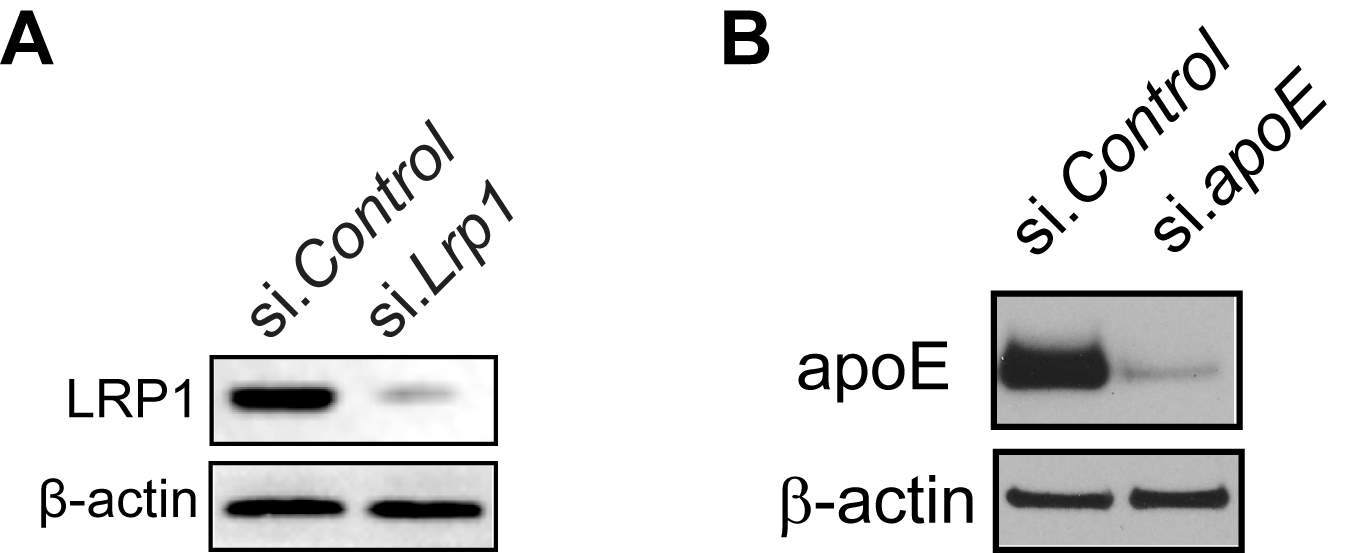
**

**(A)** Western blot of LRP1 level in cultured pericytes at 24 h after si.*Control* or si.*Lrp1* transfection. Actin is used as a loading control. **(B)** Western blot of apoE in cultured pericytes at 24 h after si.*Control* or si.*apoE* transfection. Actin is used as a loading control.
